# The space paradox in graphic representation

**DOI:** 10.3389/fpsyg.2022.968918

**Published:** 2022-10-04

**Authors:** Christiane Lange-Küttner, Ximena Vinueza Chavez

**Affiliations:** ^1^Department of Psychology, University of Bremen, Freie Hansestadt Bremen, Germany; ^2^School of Psychology, London Metropolitan University, London, United Kingdom

**Keywords:** visual realism, object-based attention, space-based attention, drawing development, negative space technique, visual attention, spatial concepts, 3D rendering

## Abstract

The negative space drawing technique refers to drawing the transparent space around and between objects, rather than drawing the objects themselves. This space-based instruction is thought to attenuate object-specific visual attention and to enhance perception of a spatial expanse. Developmentally, it is equivalent to the Piagetian dichotomic space concept of filled and empty space. A sample of 96 children from 5 to 12 years of age and 24 adults (*N* = 120) drew on a computer tablet a real-life model spacebox placed in front of the participant, with three cubes placed inside the model. Children followed two instructions, a *Visual Realism (VR) Instruction* “Please draw the three cubes and the box as you can see them” and a *Negative Space (NSp) Instruction* “Please draw the space around the objects,” with the sequence counterbalanced. NSp outline drawings began to show from 9 years onwards. A positive effect of the NSp technique showed for occlusion drawing because of the depiction of common contour of objects which could create a cohesive scene feature such as a horizon. The VR instruction focused attention toward the space box and enhanced 3D drawing of both the spacebox and the cubes. Thus, it could be concluded—rather paradoxically—that drawing in 3D is better based on object- than on space-based attention, while drawing occlusion is better based on space-based than object-based attention. We suggest, however, that a better definition of VR as attention to object appearances is that VR unifies objects and spatial context into one global plane.

## Introduction

Object-based and space-based visual attention differ from each other insofar as attention is biased either toward object shapes or toward locations that are distributed in space (Beck and Kastner, 2014). Adults are able to devise either kind of attention depending on the task affordances. For instance, in an apparent motion task two stationary objects when presented at a critical interval can be perceived as moving from A to B. This illusory movement perception should employ space-based attention, however, when the instruction was to compare features of the two stationary objects, object-based attention occurred (Zheng and Moore, 2021). Surprisingly, this well-established terminology is not in use in developmental psychology, with PsycInfo showing only one study that is using the concept of object-based vs. space-based attention in its abstract (Valenza and Calignano, 2021). This is the more astonishing because there is a clear transition in the graphic representations of children from object-based to space-based constructions (Lange-Küttner, 2008a, 2020). What reliably occurs in drawing development is that young children depict just objects in implicit space, while older children make the spatial context explicit by depicting areas and perspective. The theory for this development goes back to Luquet (1927/2001) and Piaget and Inhelder (1956) who analyzed the degree of visual likeness in terms of realism. They assumed that young children draw what they know about objects which they termed “intellectual realism” resulting in fairly schematic drawings of a technical and often minimalistic character (Lange-Küttner et al., 2002), while older children draw their optical impressions and appearances, that is, they would draw what they see, termed “visual realism” (VR). Intellectual realism was shown to be due to a deeply entrenched attitude as children would create the same kind of drawings during immediate repetitions (Lark-Horovitz, 1941) and even after years (Lange-Küttner, 1994). If something happened during practice, it was that children would lose out in details during repetitions, only to be temporarily saved by a new drawing theme, but sometimes they would even regress to earlier stages of realism (Lange-Küttner et al., 2014). Hence developmental psychologists began to explore how children's mental mindsets could be swayed toward more advanced ways of depiction.

How flexible the drawing rules of children would be was first ascertained by giving children half-finished drawings. The early tadpole drawings of children are created by just drawing a circle with a face and adding “arms” and “legs” to this circle. Hence, these human figures had arms coming out of their heads (Freeman, 1975). However, when two circles were presented ready-made as a start, one for the head and one for the trunk, and children just had to add the extensions, they would not add them to the head, but correctly to the trunk. The use of incomplete drawings proved to be a very successful and replicable technique (e.g., Boyatzis et al., 1995). Another way of testing mental flexibility when drawing was to give different instructions. For instance, when children were asked not just to draw a human figure, but to draw a person that does not exist, the younger children would eliminate parts, while the older children would insert parts from different types of objects and modify the actual shape, which is a strategy that is also important in visually realistic drawings (Karmiloff-Smith, 1990). Also this method proved to produce reliable results in follow-up research (e.g., Berti and Freeman, 1997; Picard and Vinter, 1999). Thus, there are techniques that are feasible to both getting children ahead, and to reveal the mechanisms behind different drawing stages and styles.

Also with regards to the drawing of space in three dimensions on a two-dimensional drawing surface, research has produced reliable and replicable results. Young children would draw objects floating in empty space even when in located in a real-life spatial context (Dillon, 2022). Nevertheless, they do conserve not only left-and-right placements, but also depth as objects behind each other are drawn along an implicit vertical axis (Light and MacIntosh, 1980). This can be explained with their knowledge of topological relations between objects (Piaget and Inhelder, 1956). Especially in their work on distance, an experiment showed that children claim that the distance between A and B is reduced when a third object C is inserted (Piaget et al., 1960). This proved the dichotomous quality of topological space, one the one hand space being filled by objects, on the other hand space being an empty and transparent intermedium (Piaget et al., 1960). The topological concept is comparable to solid-state physics in astronomy where objects are floating in the infinite expands of deep space (Plummer et al., 2016; Bower and Liben, 2021). In fact, this notion was picked up in early pedagogy going back to Goethe (Clarke, 1912) and Steiner (Uhrmacher, 1995) who encouraged the teaching of a cosmic perspective where orientation and self-evaluation in space and the universe would lead to spatial exploration and modesty. Modesty appears to be also reflected in children's drawings of spatial systems where the average size of the human figure shrinks, the more explicit the spatial axes system becomes (Lange-Küttner, 1997, 2004, 2009). Piaget (1955) termed this process “de-subjectivation” as children would consider themselves as just another object in space which would lead to an increased ability to modify their own actions in response to failure and create an opportunity to optimize plans and strategies. With regards to drawing, it is the ability for size modification that develops, not just size reduction, as the human figure can be a point in space, or be depicted in an oversized portrait (Lange-Küttner, 2008b).

**Figure 1:**
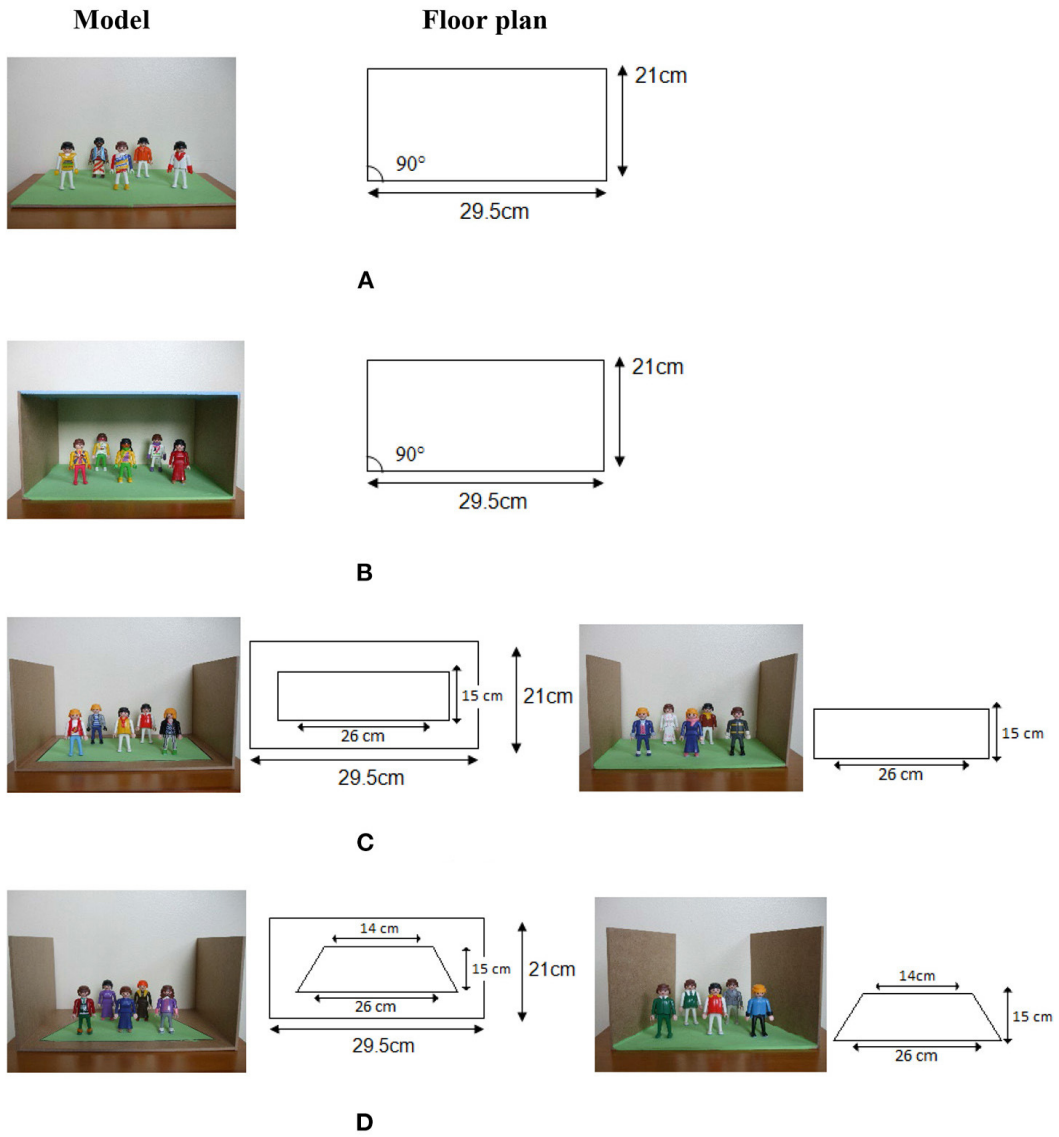
Models simulating pictorial space concepts. The floor plan gives information about the objective measurements in cm. In **(B–D)**, the walls on either side were 15 cm high. In **(C)** the ground plan is orthogonal, while in **(D)** the ground plan is trapezoid. **(A)** Empty space. **(B)** Earth space (heaven). **(C)** Playing field boundaries. **(D)** Trapezoid built-in perspective. Figures reproduced with friendly permission of the American Psychological Association (APA) (Lange-Küttner, 2014).

The relationship between intellectual and visual realism in the drawing of pictorial space was further explored with 3D models that simulate the development of spatial systems in children's drawings (Lange-Küttner, 2014; see Figure 1). Even young children aged 4 would draw walls of small spatial models (Dillon, 2022); the ones in Figure 1 were used in a drawing experiment with children between 7 and 11 years of age (Lange-Küttner, 2014).

Model 1A resembles the implicit empty space of young children's drawings. No walls or delineated fields constrain the empty expanse. Model 1B emulates the “air gap” drawings of children who draw groundline and skyline with horizontal spatial axes (Hargreaves et al., 1981; Cox and Chapman, 1995). Children denote with these stripes that one can walk on the ground due to gravity, there is a blue-colored heaven above, and in between, there is transparent air. The two models in Figure 1C do not show an air gap anymore. Instead, an area with explicit rectangular spatial boundaries is constructed. The only difference in Figure 1D is that the sides of the rectangular field converge so that the spatial field is a trapezoid. Note that while the ground plan reveals this difference, the photographic images of the space boxes show converging lines at every level, representing the optic impression. Thus, only in model 1D is the ground plan in agreement with the optically correct photographic image. This model lead children as young as 7 years old to sketch the diagonals of perspective, and even more often than 9- to 10-year-olds, while normally, perspective drawings only emerge in the drawings of older children, and also only in a minority of adults (Hagen, 1985).

## The current study

However, although the development of the space concept is usually understood space-based, three-dimensional depth can also be constructed by drawing overlapping objects, that is, object-based. In order to do this successfully, children need to learn a new technique which has been called “hidden line elimination” as the object in the front will interrupt the contour of the object behind as only a partial view would be visible. Thus, parts of the occluded object shape need to be omitted. Instead, the figure would have a shape with an open and incomplete outline. However, young children would draw occluded objects either separately, or transparent just drawing one shape over the other (Morra et al., 1996). The developmental problem here is that on the one hand, children find it hard to draw an incomplete rather than a whole object (Lange-Küttner, 2000), on the other hand, a perceptual aspect is that they have to be good in detecting the outline of a shape as for instance in visual noise in the Embedded Figures Test (EFT, Witkin, 1950; Lange-Küttner and Ebersbach, 2013). A cognitive factor is that working memory has to be mature in order to cope with the various aspects of drawing occlusion, for instance, children find it confusing if the occluded object has the same shape as the one in front (Morra, 2002).

**Figure 2:**
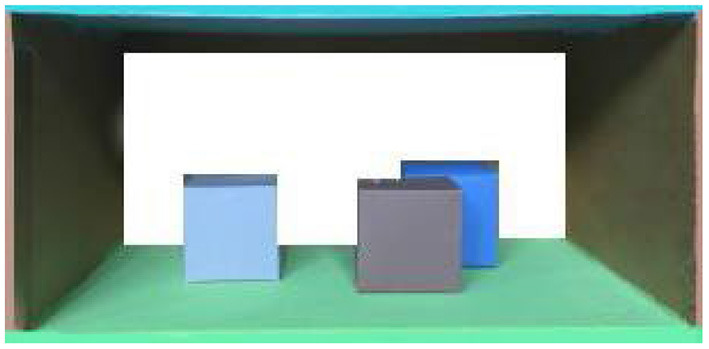
The drawing model.

Importantly, longitudinal research showed that depth in drawings is first created object-based, using occlusion of objects, followed by the unfolding of the third dimension in the whole of the pictorial space (Lange-Küttner, 1994). In order to test whether children draw object-based occlusion or space-based perspective, we devised in the current study a model that closely matches previous experimental research (Lange-Küttner, 2014). However, the earth model was not populated by visually isolated figures, but by one single and two overlapping cubes (see Figure 2). We selected the earth model (Figure 1B) as it should appeal to the topological notion of space consisting of solid objects in transparent air.

It has been claimed that object knowledge and especially object labels would actually hinder drawing in perspective (Edwards, 1992). There is some evidence that it is true that nonsense objects are less likely to trigger schematic and holistic drawing templates than meaningful figures in children (Tallandini and Morassi, 2008). Both handling and naming objects prevented visually realistic occlusion (Bremner and Moore, 1984). Also knowledge of the true object size can be an obstacle for the depiction of projective size (Reith and Liu, 1995). Thus, object knowledge can indeed inhibit the ability to draw object-based depth which has been evaluated as the suppression of a sensory core (Costall, 1995).

**Figure 3:**
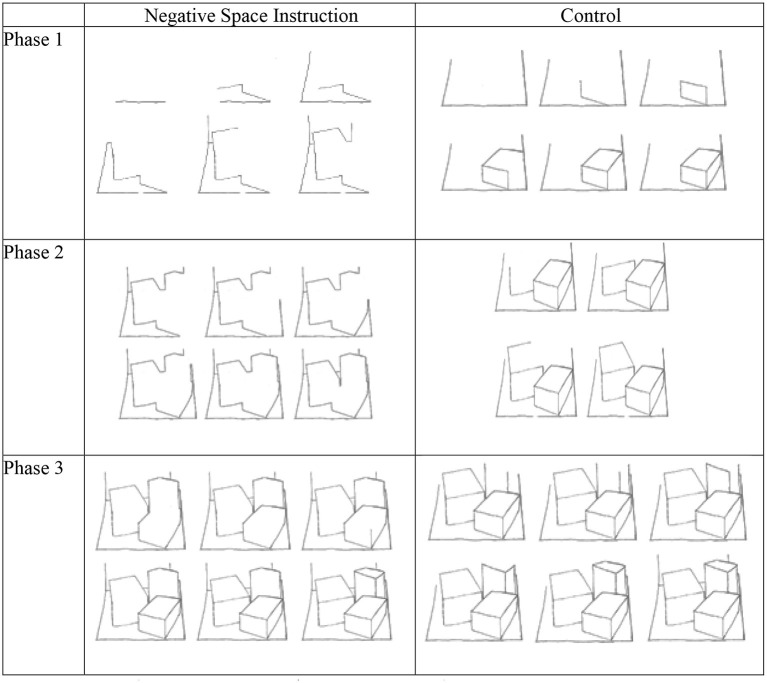
Effects of the negative space (NSp) instruction on drawing cubes. See Nunn (2009), p. 199–203, Figures 10.1–10.6.

To inhibit the focus on objects in order to foster space-based drawing, the negative space (NSp) technique was suggested (Edwards, 1987). This technique requires to draw the transparent space (“intermedium”) between objects rather than the objects themselves. Since the transparent air ends where an object begins, there is a shared boundary which quasi-automatically will reveal the objects. This idea has been empirically tested with adults drawing three overlapping cubes on a carpet (Nunn, 2009). While the negative space technique did not bestow any advantages on the draftsman as the final outputs were very similar, the actual process of drawing was fundamentally changed from object-based to a space-based attention, especially in phases 1 and 2, but not toward the end (see Figure 3).

In the control condition the carpet was drawn first, then the three boxes one by one until the drawing was completed. In the NSp condition, participants first drew the negative space around the objects which coincided with the outer contours of all three boxes as a group and then proceeded to draw the inner edges.

Could this NSp technique also be used with children when drawing overlapping cubes in a space box? Based on previous research, we predicted that when following the negative space instruction, the occluded cubes (object-based depth) would be drawn in a less mature fashion than with the visual realism instruction, while the overall space of the earth model would be depicted in a more advanced 3D fashion (space-based depth).

## Methods

### Participants

We randomly recruited 120 participants from London (UK) schools. The age in years; months for each age group is listed in Table 1, with 12 females and 12 males in each group. Participants had full or corrected vision. Children with special educational needs (SEN) who were allocated a personal teaching assistant did not participate in the experiment.

**Table 1:** Age groups (years; months).

**Age in years**	***M* (years; months)**	***n***	**Min**	**Max**	***SD***
5–6	5; 6	24	63	71	3
7–8	7; 7	24	88	96	3
9–10	9; 7	24	111	120	3
11–12	11; 5	24	132	143	4
Adults	32; 5	24	240	540	89
Total		120			

### Apparatus and materials

The floor and the heaven of the spatial drawing model (see Figure 2) were 29.5 × 21 cm in size. The walls on either side were 15 cm high. The model contained three plastic cubes (brown, blue, and gray) each 5.5 × 5.5 × 5.5 cm in size, one single cube and two overlapping cubes.

Drawing was carried out with a stylus pen on a convertible Lenovo Yoga tablet/laptop with a Windows 10 system. The size of the screen was 13.3 inches. Windows Paint Software and Icecream Screen Recorder Software, version 370 Pro, made it possible to capture the area of the screen as a video file.

### Procedure

The ethics proposal of the study was approved by the London Metropolitan University departmental Ethics Committee. Parents of children were given information sheets and consent forms. Only those children who brought signed consent forms from their parents to school were actually tested. All participants were also asked whether they were happy to take part in the study immediately before the start of the experiment.

In order to test two children at the same time, the equipment was doubled up, that is, there were two drawing models and two convertible laptop/tablets. In a classroom, two tables were allocated, separated, and lined up along a wall so participants were not able to see each other. Each table with one spatial model and cubes was set up in advance. Once the setup was ready, the participants were seated on a chair in front of the model that was placed at a distance of about 40 cm from the participant. The participants were randomly allocated to one of the two sequences of instructions. Sixty of the participants started drawing under the visual realism instruction “Please draw the three cubes and the box as you can see them” and then under the negative space instruction “Please draw the space around the objects.” The other half of participants started in the reverse order. The laptop/tablet screen was completely white; all participants drew two pictures on it from the same viewing position. Participants were informed that they had a maximum of 10 min per drawing.

### Data generation

The drawings were scored by two 3rd year Architecture student as raters. The raters were blind to the children's drawing instructions. Only the evaluation criteria were explained. Occlusion and 3D volume of the cubes were rated according to an adapted version of the rating manual of Lange-Küttner and Ebersbach (2013); see Table 2. It was also scored whether children and adults were drawing outlines which were to be expected in the NSp Instruction condition.

**Table 2:** Categorization of the drawings: cube volume and occlusion.

**Cube volume**	**Description**	**Score**	**Examples**
Orthographic	One-face cube: all sides implicit	1	
			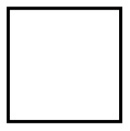
Vertical or horizontal	Two-Face Cube: Front face plus top or side face unfolded	2	
			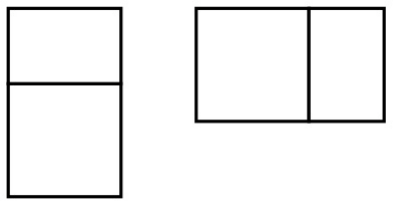
Diagrammatic, fold-out	Front face plus top and side faces unfolded (same ground line)	3	
			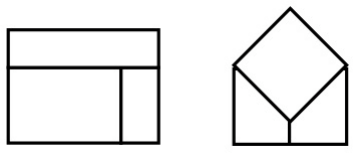
Partly wrong perspective	3D, but no parallel lines	4	
			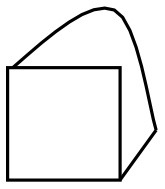
Oblique/viewpoint perspective	Oblique angles, parallel lines or common vanishing point	5	
			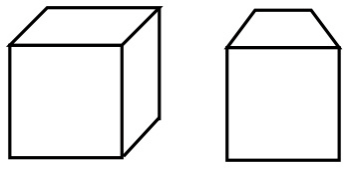
**Occlusion**			
None		0	
			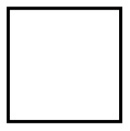 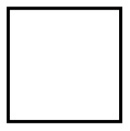
Intersection		1	
			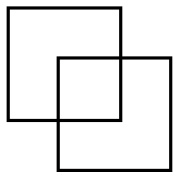
Occlusion		2	
			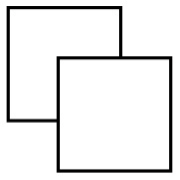
**Outline**			
Outline around one cube		1	
			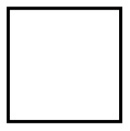
Outline around two cubes		2	
			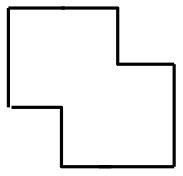
Continuous outline		3	
			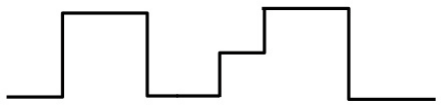

Scoring manual adapted from Lange-Küttner and Ebersbach (2013).

The space system of the spacebox itself was scored following the rating schedule of Lange-Küttner (2004, 2009, 2014), with a score of 1 for implicit space, a score of 2 for groundline and stripy images, a score of 3 for the depiction of delineated fields and a score of 4 for the depiction of the field in perspective. For the current study, this rating schedule was adapted because a playing field was not relevant. A drawing of just objects but no drawing of the space box received a score of 0, drawing just a two-dimensional frame for the box was scored with 1, a score of 2 was given for groundline and stripy images, a score of 3 was awarded if the walls at the side were drawn, and a score of 4 for the depiction of the entire spacebox in three-dimensional perspective. Agreement was 70% which is within the normal range. Disagreements between raters were solved in a discussion. From the videos, the second author rated whether the space box, or the cubes were drawn first.

## Results

We adjusted the degrees of freedom when the Mauchley's (ANOVAs) or Levene's (*T*-tests) tests for the equality of variances were significant. This correction is easily identifiable as the samples are of equal size and thus the corrected degrees of freedom clearly differ. Pairwise comparisons within the ANOVA models were corrected by SPSS using Bonferroni. Effect sizes are partial etas. Raw data are available on https://osf.io/7w5sc/.

We first controlled whether the negative space instruction worked by testing the expected outlining of grouped cubes inside the space box. Once this was confirmed, we analyzed the overall space system of the drawings, followed by occlusion and volume of the cubes, and the scores resulting from the video analysis showing whether the box or the cubes were drawn first.

### Outline

**Figure 4:**
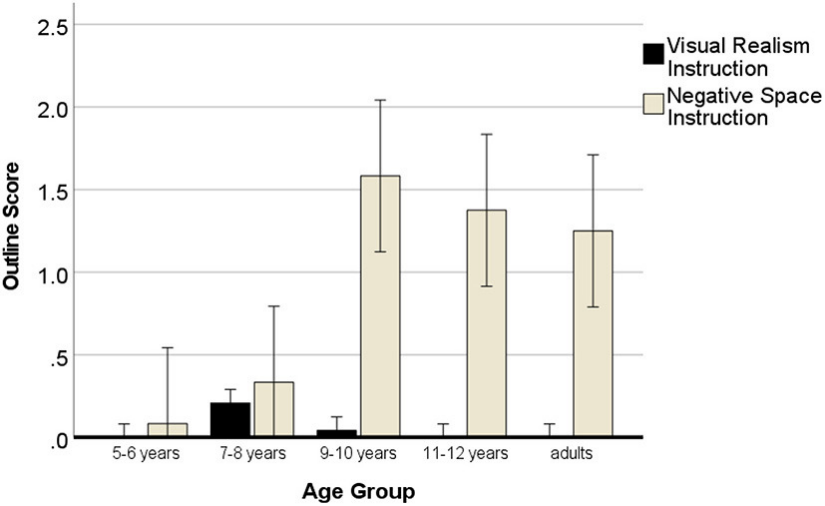
Development of outline drawings with negative space instructions. Error bars denote the confidence interval.

In this analysis, we expected that outline drawings would only occur in the negative space instruction condition. A 5 (age group) × 2 (instruction) × 2 (sequence) ANOVA with repeated measures for the outline score showed no effect of the sequence of the instruction, *p*_*s*_ > 0.478. There was a main effect of age, *F*_(4, 120)_ = 7.36, *p* < 0.001, η^2^ = 0.21, a main effect of instruction, *F*_(1, 120)_ = 69.02, *p* < 0.001, η^2^ = 0.39 and a significant two-way interaction of instruction with age, *F*_(4, 120)_ = 9.12, *p* < 0.001, η^2^ = 0.25. Figure 4 shows that children and adults drew outlines almost only in the NSp instruction condition.

Paired samples *t*-tests (one-tailed) of outline drawings (Table 3) after the two types of instructions showed the negative space instruction was leading to a significant increase in the expected outlines of cubes in the NSp instruction from 9 years onwards. This confirmed our expectation that the negative space instruction could also be used with children.

**Table 3:** Paired samples *t*-tests of outline drawings after VR and NSp instructions.

**Age groups (years)**	***r***	**Cohen's d**	***t***	***df***	***p***	**95% CI**	
						**Lower bound**	**Upper bound**
5–6	−	−0.204	−1.000	23	0.164	−0.606	0.202
7–8	−0.14	−0.184	−0.901	23	0.188	−0.586	0.222
9–10	0.06	−10.115	−5.463	23	0.001**	−1.620	−0.595
11–12	−	−0.976	−4.783	23	0.001**	−1.458	−0.480
Adults	−	−0.879	−4.307	23	0.001**	−1.345	−0.399

**p < 0.01, r = – (correlations not computed because of floor effect).

### Space system

We analyzed whether the two drawings of the space system showed the expected development with age and an effect of instruction with a 5 (age group) × 2 (instruction) × 2 (sequence) ANOVA with repeated measures for the drawing instruction of the space system. The sequence of the instruction was not important, *p*_*s*_ > 0.165. Age group showed a significant effect, *F*_(4, 120)_ = 17.30, *p* < 0.001, η^2^ = 0.39. Even the 5- to 6-year-olds drew the box with a frame (*M* = 1.08), but they significantly differed from all other age groups, *p*_*s*_ < 0.002, who constructed more advanced spatial systems. The 7- to 8-year-olds (*M* = 2.04) and the 9- to 10-year-olds (*M* = 2.37) drew groundlines and stripy pictures, but differed from the adult group who drew the walls of the spacebox (*M* = 3.10), *p*_*s*_ < 0.043, but not from each other. The 11- to 12-year-olds (*M* = 2.42) did not differ significantly from the adult group.

**Figure 5:**
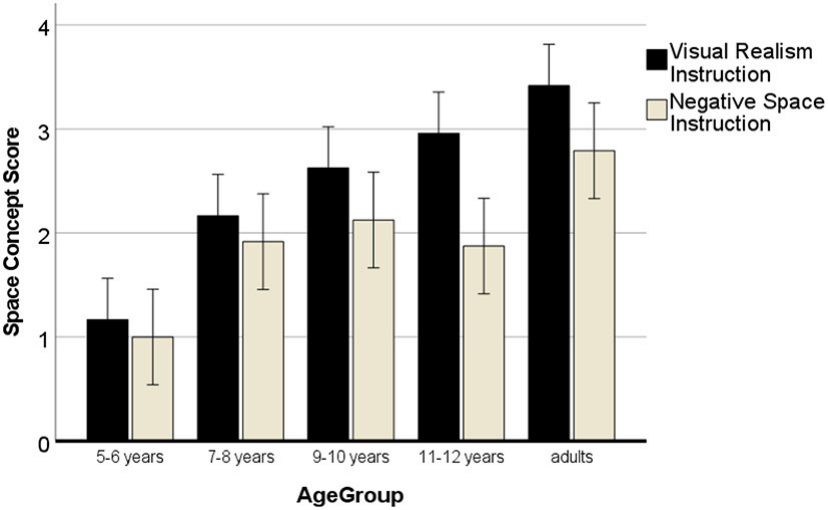
Development of the 3D space system in drawings with negative space instructions. Error bars denote the confidence interval.

Importantly for the hypothesis, the effect of instruction was significant, *F*_(4, 120)_ = 21.43, *p* < 0.001, η^2^ = 0.16, but the interaction with age was only a trend and did not reach significance, *p* < 0.093. Figure 5 shows that the VR instruction yielded more advanced space systems in every age group.

However, pairwise comparisons (one-tailed) of the space system of the two drawings showed that the difference was only significant from 9 years onwards (see Table 4). The high correlation between the two drawings in the 5- to 6-year-olds indicates that the youngest children did not make much difference because of the instructions.

**Table 4:** Paired samples *t*-tests comparing 3D space systems after VR and NSp instructions.

**Age groups (in years)**	***r***	**Cohen's d**	***t***	***df***	***p***	**95% CI**	
						**Lower bound**	**Upper bound**
5–6	0.65**	0.22	1.072	23	0.147	−0.188	0.622
7–8	0.17	0.21	1.030	23	0.157	−0.197	0.613
9–10	0.38*	0.40	1.958	23	0.031*	−0.021	0.812
11–12	0.30	0.77	3.760	23	0.001**	0.304	1.218
Adults	0.17	0.42	2.044	23	0.026*	−0.005	0.813

*p < 0.05; **p < 0.01.

### Occlusion

The same model of variance was used to test whether occlusion would differ according to instructions. Drawing of occlusion increased with age, *F*_(4, 120)_ = 30.83, *p* < 0.001, η^2^ = 0.53, with a higher effect size than for the space concept. The score of the 5- to 6-year-olds was close to zero drawing spatially isolated cubes (*M* = 0.21), and again they significantly differed from all other age groups, *p*_*s*_ < 0.003, except for the 11- to 12-year-olds, *p* = 0.081. The 7- to 8-year-olds showed the best performance of the children's groups (*M* = 1.02) and significantly differed from the youngest (*M* = 0.21), the 11- to 12-year-olds (*M* = 0.58) and the adults (*M* = 1.67) whose score was closest toward the complete overlap score of 2. Different to the 3D space concept, there was not a continuous gradual increase in the occlusion score.

In this model, the sequence was important for the instruction, *F*_(1, 120)_ = 4.16, *p* = 0.044, η^2^ = 0.04, and sequence interacted with age, *F*_(4, 120)_ = 3.13, *p* = 0.018, η^2^ = 0.10, but the three-way interaction was not significant, *p* = 0.404. Pairwise *t*-tests (two-tailed) per sequence group showed that when the visual realism instruction was given first, occlusion was drawn in the same way as with the NSp instruction (VR *M* = 0.75; NSp *M* = 0.82, *r* = 0.47^***^) without a significant difference, *p* = 0.542. However, when the NSp instruction was given first, drawing of occlusion was improved (VR *M* = 1.03; NSp *M* = 0.77, *r* = 0.37^**^), *t*_(59)_ = 2.21, *p* = 0.031, showing that most participants would draw at least intersecting cubes.

**Figure 6:**
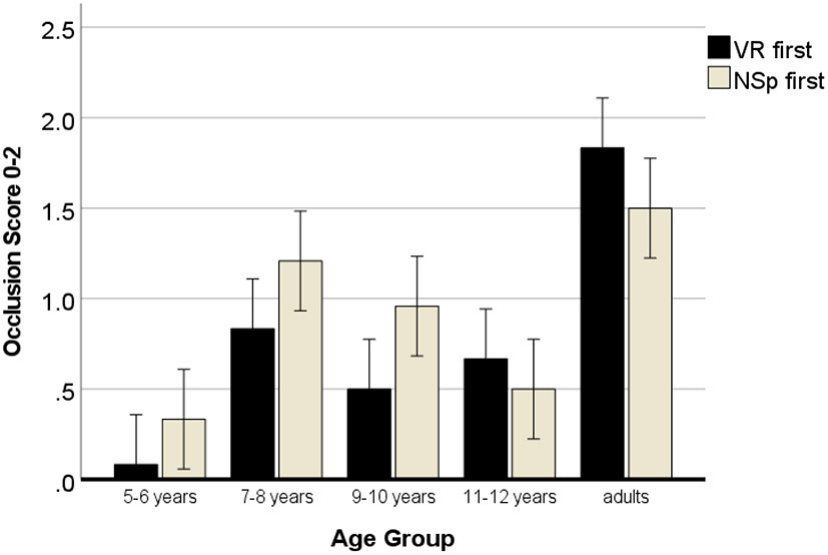
Development of drawing occlusion with negative space or visual realism instructions. Error bars denote the confidence interval.

The two-way interaction of sequence with age groups is visualized in Figure 6. It shows that the 5- to 10-year-old children were more likely to attempt to draw the cubes overlapping when they first were asked to draw the space between the objects, rather than to draw what they see, while the 11- to 12-year-olds and the adults were more likely to attempt to draw occlusion when first being asked to draw what they see. The results of the *t*-tests for independent samples (one-tailed) in Table 5 reveal medium effect sizes but relatively low *p*-values, while the change in sign of the *t*-value denotes the interactive effect.

**Table 5:** Independent samples *t*-tests comparing occlusion after VR or NSp instruction FIRST.

**Age groups (in years)**	**Cohen's d**	***t***	***df***	***p***	**95% CI**	
					**Lower bound**	**Upper bound**
5–6	−0.67	−1.636	14.36	0.062	−0.577	0.077
7–8	−0.69	−1.704	22	0.051	−0.831	0.081
9–10	−0.83	−2.030	22	0.027*	−0.927	0.010
11–12	0.41	1.000	22	0.164	−0.179	0.512
Adults	0.66	1.609	16.34	0.063	−0.105	0.772

*p < 0.05.

### Volume

**Figure 7:**
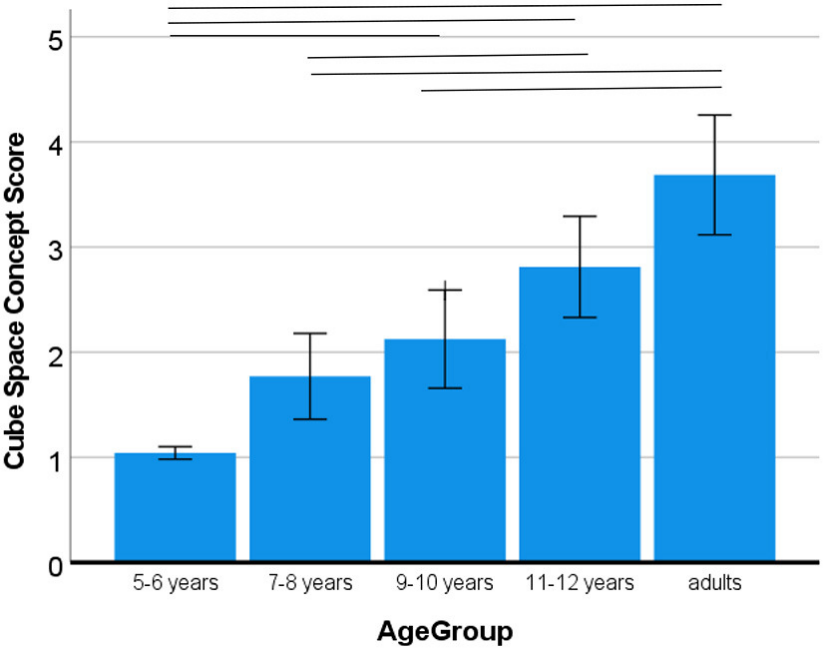
Development of drawing cubes in 3D. Error bars denote the confidence interval. Horizontal bars indicate significant pairwise comparisons.

While we did not have a hypothesis about the effect of the VR and the NSp instruction on the three-dimensional volume of the cubes inside the earth spacebox, we still wanted to control whether there was an effect. Hence, the same model of variance was used to test whether cube volume would differ according to instructions. Like for the volume of the space box, the sequence of instructions did not play a role for the three-dimensional volume of the cubes, *p*_*s*_ > 0.225. A main effect of age, *F*_(4, 120)_ = 23.31, *p* < 0.001, η^2^ = 0.46, showed a pronounced increase in the depiction of the third dimension of cubes (best score) with age (5–6 years: *M* = 1.04; 7–8 years: *M* = 1.77; 9–10 years: *M* = 2.12; 11–12 years: *M* = 2.81; adults: *M* = 3.69; see Figure 7). As there were many significant pairwise comparisons, all clearly indicating significant progression, these are indicated in the figure and not further explained here.

**Figure 8:**
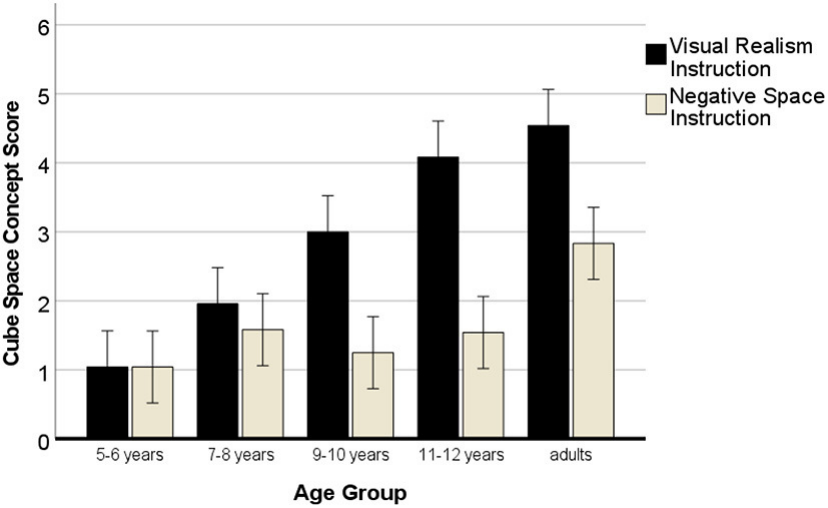
Development of drawing cubes in 3D with negative space or visual realism instructions. Error bars denote the confidence interval.

Moreover, there was a significant main effect of the drawing instruction, *F*_(1, 120)_ = 78.91, *p* < 0.001, η^2^ = 0.42, and a two-interaction of instructions with age, *F*_(4, 120)_ = 10.81, *p* < 0.001, η^2^ = 0.28 (see Figure 8). The main effect showed that the visual realism instruction yielded more multi-dimensional cube drawings (*M* = 2.92) than the negative space instruction (*M* = 1.65). However, the two-way interaction with age demonstrated that this effect increased with age, the older the participants, the more efficient was the instruction to draw what they were seeing for drawing three-dimensional cubes, and the larger the difference in efficiency to the negative space instruction in this regard.

The results of the pairwise *t*-tests (one-tailed) for the two 3D cube drawings in each age group in Table 6 reveal that the higher efficiency of the visual realism instruction in yielding multi-dimensional cube drawings becomes significant from 9 years onwards.

**Table 6:** Paired samples *t*-tests comparing 3D cube volume after VR and NSp instructions.

**Age groups (in years)**	***r***	**Cohens' d**	***t***	***df***	***p***	**95%** ***CI***	
						**Lower Bound**	**Upper Bound**
5–6	−0.04	0.00	0.000	23	0.500	−0.400	0.400
7–8	0.18	0.23	1.141	23	0.133	−0.175	0.636
9–10	0.33	1.04	5.120	23	0.001**	0.537	1.538
11–12	0.25	1.44	7.040	23	0.001**	0.854	2.004
Adults	0.36*	0.88	4.304	23	0.001**	0.399	1.345

**p < 0.01.

### What was drawn first, the space box or the cubes?

Participants had only the choice to either start drawing the space box, or drawing the cubes. Hence, these two alternatives are linked insofar as if the drawing was started with depicting the space box, the cubes were not drawn first. Hence, it was tested with the same model as before whether the spacebox was drawn first as this would speak to a space-based approach. The sequence of the instructions was not important, *p*_*s*_ > 0.297. There was a main effect of age, *F*_(4, 120)_ = 9.06, *p* < 0.001, η^2^ = 0.25 (5–6 years: *M* = 0.62; 7–8 years: *M* = 0.90; 9–10 years: *M* = 0.94; 11–12 years: *M* = 0.92; adults: *M* = 0.62), which showed that the 5- to 6-year-old children were less likely to start their drawing with an outline of the space box than any other age group of children, *p*_*s*_ < 0.005, but with the same likelihood as adults.

**Figure 9:**
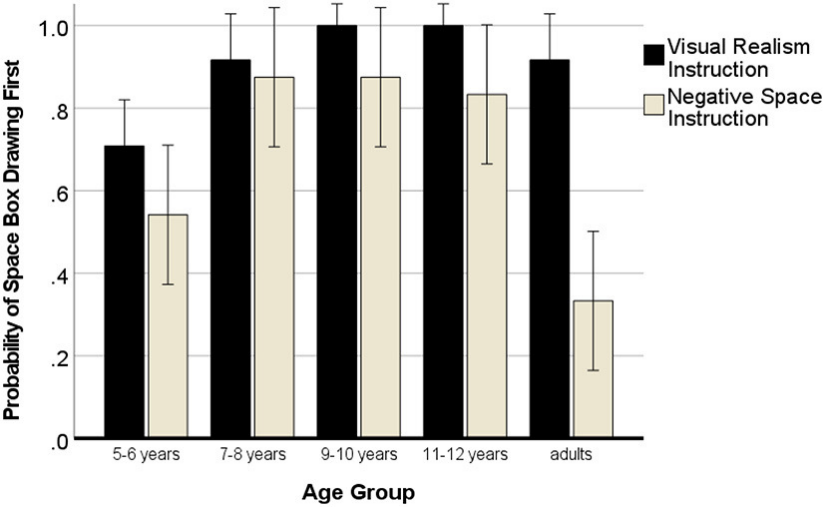
Probability of drawing the space box first (space-based approach). Error bars denote the confidence interval.

There was a significant effect of instruction, *F*_(1, 120)_ = 24.88, *p* < 0.001, η^2^ = 0.18 showing that the visual realism instruction yielded more drawings that were started with the space box depiction (VR *M* = 0.91; NSp *M* = 0.68), however, the extent of the effect of the VR instruction varied with age *F*_(4, 120)_ = 4.71, *p* = 0.002, η^2^ = 0.15 (see Figure 9).

The results of the pairwise *t*-tests (one-tailed) for the two drawings start scores in each age group in Table 7 reveal that the higher efficiency of yielding multi-dimensional cube drawings with the visual realism instruction becomes significant from 9 years onwards.

**Table 7:** Paired samples *t*-tests comparing occlusion drawing after VR and NSp instructions.

**Age groups (in years)**	***r***	**Cohen's d**	***t***	***df***	***p***	**95%** ***CI***	
						**Lower bound**	**Upper bound**
5–6	0.15	0.26	1.282	23	0.106	−0.148	0.666
7–8	0.34*	0.12	0.569	23	0.287	−0.287	0.516
9–10	–	0.37	1.813	23	0.041*	−0.048	0.780
11–12	–	0.44	2.145	23	0.021*	0.014	0.853
Adults	−0.11	1.0	4.897	23	0.001**	0.500	1.485

*p < 0.05, **p < 0.01, r = – (correlations not computed because of ceiling effect).

## Discussion

The current study investigated whether the negative space (NSp) drawing technique could also be used with children. We used an earth model space box where heaven was symbolized with a blue lid alluding to the stripey air gap pictures that children draw until they are about 11–12 years old when they draw the sky down to the horizon (Lewis, 1990). The gap would contain transparent air and this kind of drawing is in agreement with a dichotomous topological space concept of empty space with solid objects. As such, the earth model space box lent itself to the NSp drawing technique which requires to draw the space between objects rather than the objects themselves (Edwards, 1987, 1992). From research with adults, it had become clear that this instruction changed the drawing process as empty silhouettes were drawn first and internal features were added last (Nunn, 2009). Children are able to draw empty silhouettes, although only a minority would do so spontaneously (Reith, 1988). Thus, we expected that children would be able to draw an outer contour around overlapping cubes. We predicted that with the NSp instruction, the occluded cubes (object-based depth) would be drawn in a less mature fashion because visual attention would be directed away from individual objects. We furthermore expected that the NSp instruction would direct attention toward the overall space of the earth model that would then be depicted in a more advanced 3D fashion (space-based depth). We contrasted the NSp instruction with the visual realism (VR) instruction that explicitly requires children and adults to draw what they see. However, the VR instruction is not drawing visual attention to the intermediate space between objects, and thus away from objects, but it draws attention to the optical impression of object appearances. In this way, both types of instruction direct attention away from object knowledge, for instance, thinking about object built and function, or object labels.

### Development of 3D depth depiction

**Figure 10:**
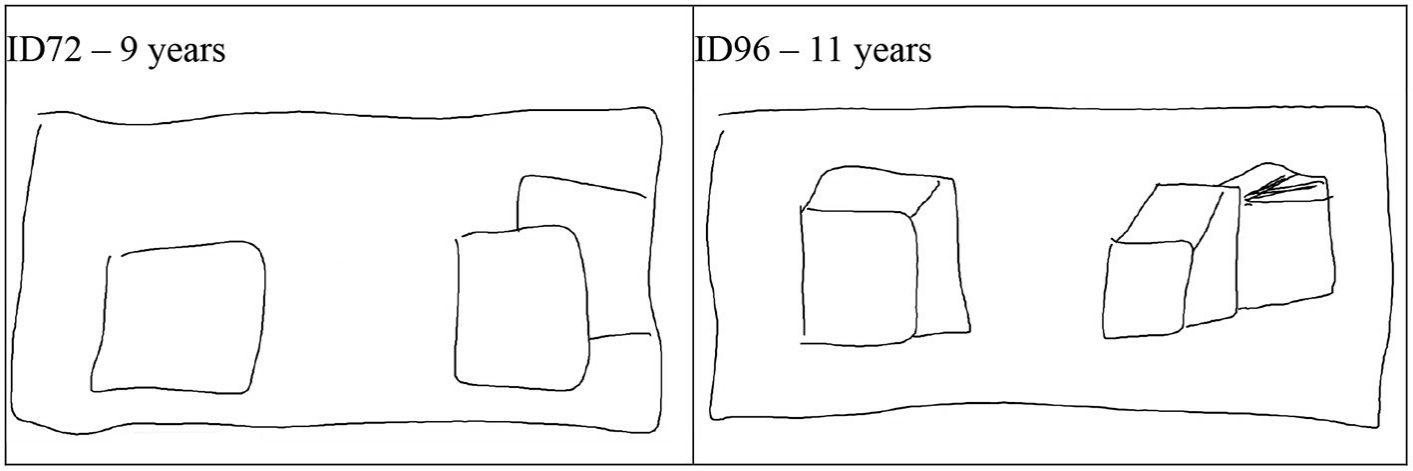
Examples of drawing occlusion with 2D and 3D cubes. ID72 - 9 years (See the film clip on https://osf.io/5b4sf/); ID96 - 11 years (See the film clip on https://osf.io/g78xv/).

We found that until 8 years there was little evidence that children would draw outlines of the air between objects rather than an object itself. However, from age 9 onwards these outlines did appear with the NSp instruction and, in accordance with our expectations, not when drawing following the visual realism instruction. The three-dimensional space system of both the space box and the cubes developed well with age, while the drawing of occlusion did not. A reason may have been that drawing overlapping cubes becomes much more complicated once the cubes are drawn in three dimensions rather than as squares that holistically and implicitly mean to contain the sides of the cube (Moore, 1986). Figure 10 shows how easy it is to draw overlapping squares in comparison to overlapping 3D cubes. The 11-year-old who knows how to draw 3D cubes still tries to attach the occluded cube in the same way as the 9-year-old, but is unsure on how to create the occluded cube in the third dimension.

When checking the correlations between drawing occluded cubes and 3D cubes here, we found that at a time when cubes are typically represented as holistic squares this highly correlated with occlusion (5–6 years *r* = 0.83, *p* < 0.001), while thereafter, when children learn to unfold and integrate the sides of a cube into a three-dimensional depiction, occlusion and 3D depiction had little variance in common (7–8 years *r* = 0.25, *p* = 0.239, 9–10 years *r* = −0.08, *p* = 0.713, 11–12 years *r* = −0.01, *p* = 0.957). However, in adults, occlusion and 3D depiction of the cubes were not two rather separate processes anymore as indicated by a significant correlation (*r* = 0.63 *p* < 0.001).

### The negative space instruction

With regards to the NSp instruction, children drew outlines around empty space mainly from 9 years onwards, but not at all after the visual realism instruction which is what was expected. However, for the three-dimensions of both the space box and the cubes the visual realism instruction was more conducive than the negative space instruction, again especially from 9 years onwards. This effect did not confirm the hypothesis that the negative space instruction should lead to more advanced spatial drawing systems of the space box. Instead, depth depiction was enhanced after the VR instruction for both object-based and space-based 3D dimensionality. Thus, it could be concluded—rather paradoxically—that drawing space in three dimensions is better based on object-based attention toward appearances than on space-based attention. However, one could argue that the visual realism to focus on “what you see” implies attention to the overall optical impressions and thus overcomes the topological dichotomous space concept of empty space and solid objects and merges the two in one continuous image.

**Figure 11:**
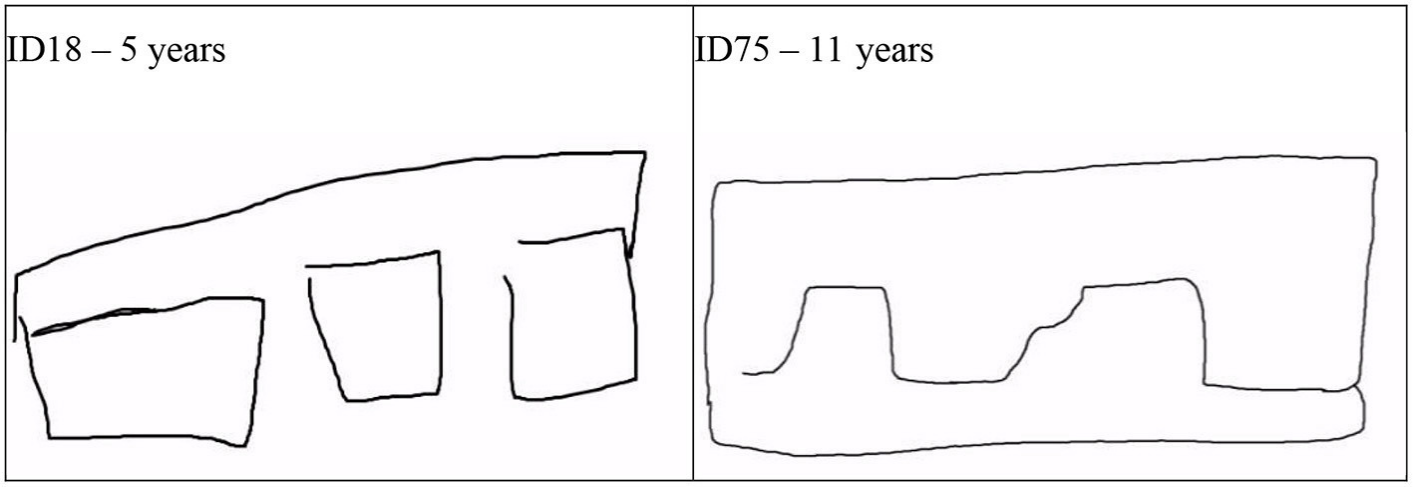
Examples of drawing negative space around occluding cubes. ID18 - 5 years (See the film clip on https://osf.io/nxw27); ID75 - 11 years (See the film clip on https://osf.io/vxe7s).

Until about 11 years, the negative space instruction tended to advance the depiction of occlusion. Also this result did not confirm the hypothesis predicting that this space-based instruction—as the air is a spatial expanse and in one of the three physical aggregate states (solid, liquid, and *aeriform*)—would lower performance in an object-based method of depth depiction such as occlusion. In occlusion, staggered and overlapping cubes are closer together than two single cubes. The NSp instruction would draw attention to common contour: Figure 11 shows the space around the three cubes drawn by a 5-year-old which looks like a bracket around the three shapes. In contrast, the 11-year-old can merge the outer contour of the cubes into what looks like a horizon line which would be part of a scene. Likewise, the same merging of the common contour of parts also occurs in the drawings of human figures at this age generating a visually realistic silhouette with a smooth outline (Lange-Küttner et al., 2002).

Visual realism is a result of the anatomy and mechanics of visual impressions. The anatomy of the brain was the model for the camera that takes photographic images. However, the brain does more than traditional cameras (modern mobile phones have two and three lenses) because it merges two visual inputs from either eye on one retinal background. This capacity to merge and transform is an essential feature of modern image software that is able to identify local objects in images, but also to merge local regions into one homogeneous pixelated image (Chen et al., 1991). In children, this decomposition and recomposition of a visual image can be mechanically facilitated by a transparent screen in front of the real objects that unifies objects and surroundings on one plane (Lange-Küttner and Reith, 1995; Reith and Dominin, 1997). Such visual operative structures were seen as essential to the epistemology of perception (Piaget, 1969).

## Conclusions

The current study makes a valuable contribution to the long-standing debate in developmental psychology on intellectual and visual realism in children's drawings as well as toward the object-based and space-based distinction of attention in cognitive psychology. We referred to earlier research showing that object-based knowledge prevents space-based visual attention that is a prerequisite for drawing visually realistic pictures. It turned out that paradoxically the apparently space-based NSp instruction enhanced object-based depth when drawing occlusion, while the apparently object-based VR instruction enhanced depiction of 3D dimensionality in both figures and context. We thus suggest that the transition from implicit to explicit space creates a new layer of a *holistic scene* that developmentally follows on from the early *holistic objects* that children draw. This notion is in stark contrast to the theory that there is a holistic-to-analytic shift in development (Kemler, 1983). Also holistic visual impressions can improve, for instance, the sure recognition of indoor vs. outdoor whole scenes improves from <20% correct at around age ten to more than 40 and up to 70% in young adults (Tang et al., 2018). Moreover, it seems that object memory vs. scene memory is modular, that is informationally encapsulated, just as intellectual realism and visual realism are deeply entrenched attitudes. In a study by Edgin et al. (2014), the scene-scene test and the object-object test were easier than a scene-object test at all ages which points to different systems. We therefore propose that future drawing research may want to compare whether the type of children's realism and what-and-where spatial memory systems develop in parallel (Lange-Küttner and Küttner, 2015).

## Data availability statement

The datasets presented in this study can be found in online repositories. The names of the repository/repositories and accession number(s) can be found at: https://osf.io/7w5sc/.

## Ethics statement

The studies involving human participants were reviewed and approved by Departmental Ethics Committee, London Metropolitan University. Written informed consent to participate in this study was provided by the participants' legal guardian/next of kin.

## Author contributions

CL-K and XVC developed the idea for this project. XVC collected the data, coordinated the ratings, and created the SPSS spreadsheet. She was awarded a Bachelor of Science degree for her project. CL-K supervised the work, wrote the text, and carried out the statistical analyses for this report. Both authors contributed to the article and approved the submitted version.

## Funding

Open-access was funded by the University of Bremen.

## Conflict of interest

The authors declare that the research was conducted in the absence of any commercial or financial relationships that could be construed as a potential conflict of interest.

## Publisher's note

All claims expressed in this article are solely those of the authors and do not necessarily represent those of their affiliated organizations, or those of the publisher, the editors and the reviewers. Any product that may be evaluated in this article, or claim that may be made by its manufacturer, is not guaranteed or endorsed by the publisher.
